# Supplementary material for: Hard time to be parents? Sea urchin fishery shifts potential reproductive contribution of population onto the shoulders of the young adults
Source: PeerJ. 2017 Mar 8;5:e3067. doi: 10.7717/peerj.3067 (PMC5345490; doi:10.7717/peerj.3067)
Supplement: Supplemental Information 1 — Results of PERMANOVA analysis on square root transformed data. P-values were obtained using 9999 Monte Carlo samples from the asymptotic permutation distribution. Significant results are in bold. [file peerj-05-3067-s001.docx]

| **Source of variation** | ***df*** | **MS** | **F** | **p** |
| --- | --- | --- | --- | --- |
| ***Time = Ti*** | 2 | 7344.3 | 7.1242 | **0.0003** |
| *Zone = Zo* | 1 | 1545.9 | 0.53823 | 0.8649 |
| *Area(Zone)= Ar(Zo)* | 2 | 1242.9 | 1.2057 | 0.3343 |
| ***Ti x Zo*** | 2 | 3544.6 | 3.4383 | **0.0108** |
| ***Ti x Ar(Zo)*** | 4 | 1030.9 | 3.1397 | **0.0001** |
| *Residual* | 24 | 328.35 |  |  |
| Pairwise tests (*Ti x Zo*)  *July ‘13*: High-pressure zone, Low-pressure zone 0.0677  *January ‘14*: High-pressure zone, Low-pressure zone 0.0668  *May ‘14*: High-pressure zone, Low-pressure zone 0.3645 | | | | |
