# Supplementary material for: Hard time to be parents? Sea urchin fishery shifts potential reproductive contribution of population onto the shoulders of the young adults
Source: PeerJ. 2017 Mar 8;5:e3067. doi: 10.7717/peerj.3067 (PMC5345490; doi:10.7717/peerj.3067)
Supplement: Supplemental Information 4 — Mean percentage (±standard deviation) of fertilized eggs and four-arm echinoplutei obtained from tests of fertility performed on US individuals from both sampling zones during the months of maximum gonadal development. na, no count of larvae was done. [file peerj-05-3067-s004.docx]

|  |  | **Dec** | **Jan** | **Feb** | **Mar** | **Apr** |
| --- | --- | --- | --- | --- | --- | --- |
| ***High-pressure zone*** | % of fertilized eggs | 90.3±5.7 | 87.3±10.4 | 92.8±4.7 | 96.0±0.8 | 91.8±6.0 |
|  | % of four-arm echinoplutei | na | 100.0±0.0 | 100.0±0.0 | 99.6±0.5 | 94.8±8.2 |
| ***Low-pressure zone*** | % of fertilized eggs | 89.8±5.4 | 93.9±0.0 | 90.5±6.1 | 92.9±0.0 | 92.3±0.5 |
|  | % of four-arm echinoplutei | na | 100.0±0.0 | 99.6±0.5 | 99.3±0.0 | 98.6±2.1 |
