# Supplementary figures and images for: Hard time to be parents? Sea urchin fishery shifts potential reproductive contribution of population onto the shoulders of the young adults
Source: PeerJ. 2017 Mar 8;5:e3067. doi: 10.7717/peerj.3067 (PMC5345490; doi:10.7717/peerj.3067)

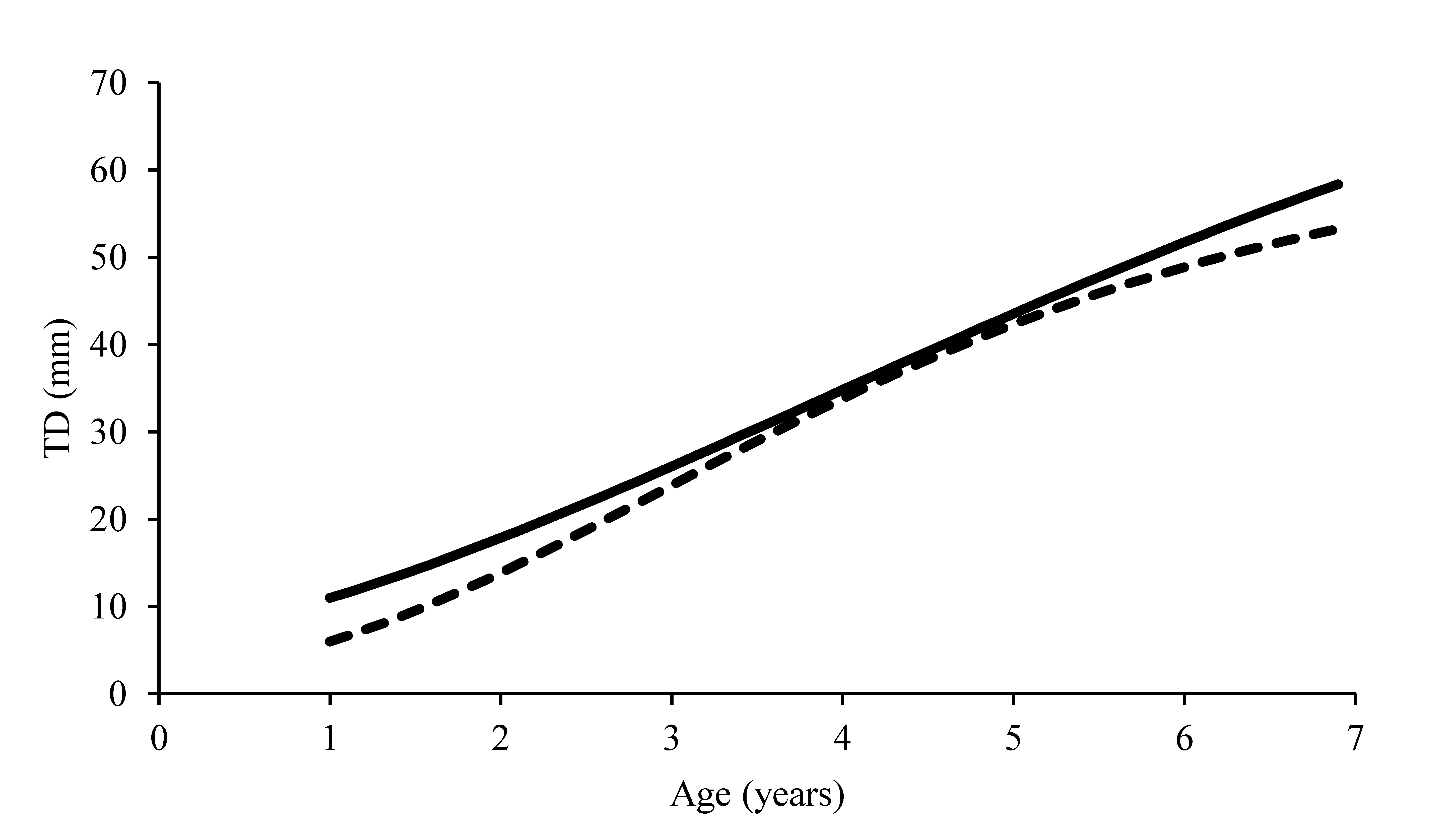

Supplement: Supplemental Information 5 — Comparison of growth rate between the populations living in the zones examined. The growth rates were compared using the non-parametric Wilcoxon test. P-value = 0.31 (n.s.). Modified from Loi et al. (2013). Dotted line, Sinis; continuous line, Tavolara—Punta Coda Cavallo. [file peerj-05-3067-s005.jpg]

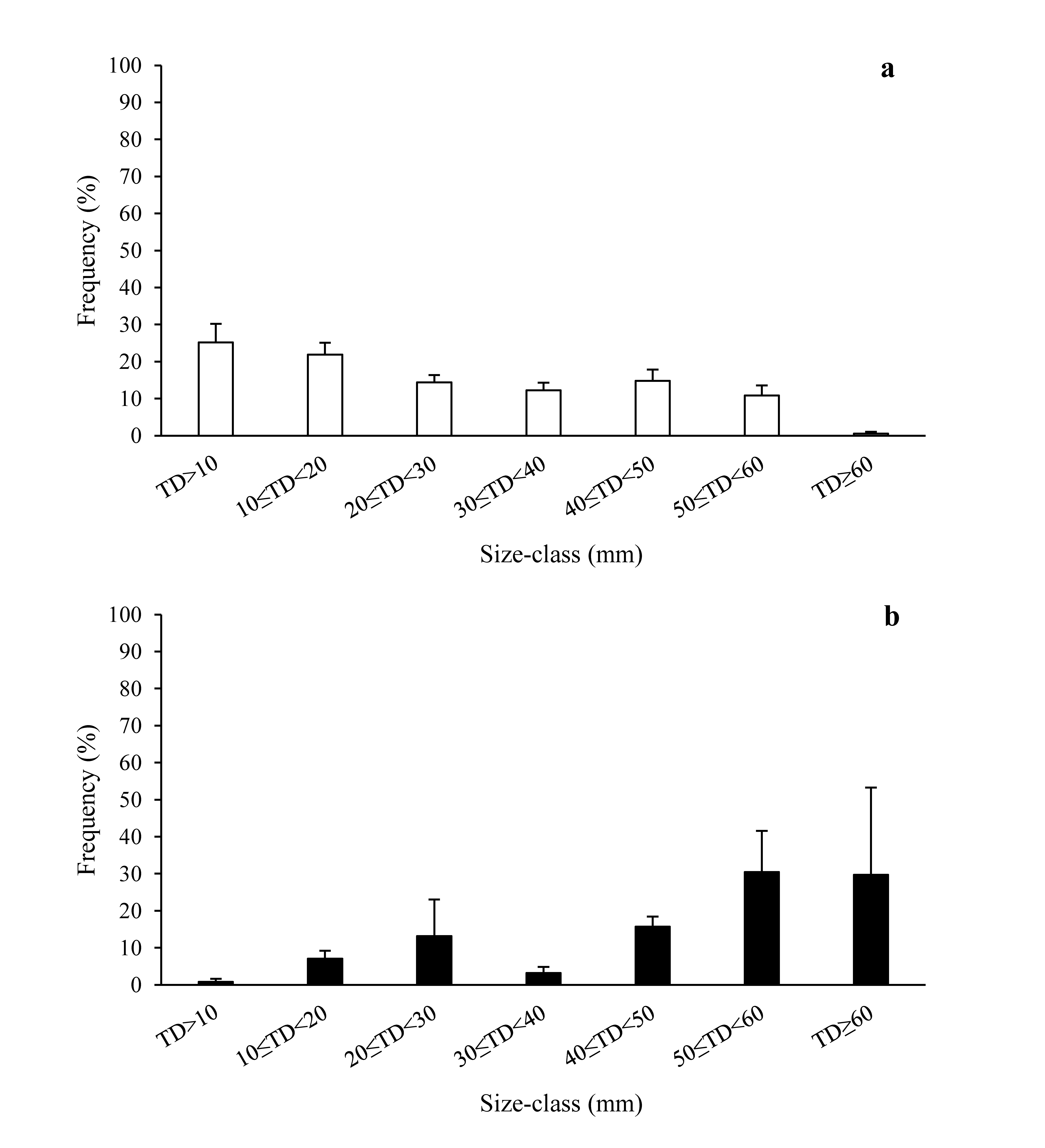

Supplement: Supplemental Information 6 — Size-frequency distribution (%) of sea urchin populations at (A) Su Pallosu Bay (Guala et al., 2006), and (B) Tavolara—Punta Coda Cavallo (Guala, Simeone & Baroli, 2009). [file peerj-05-3067-s006.jpg]

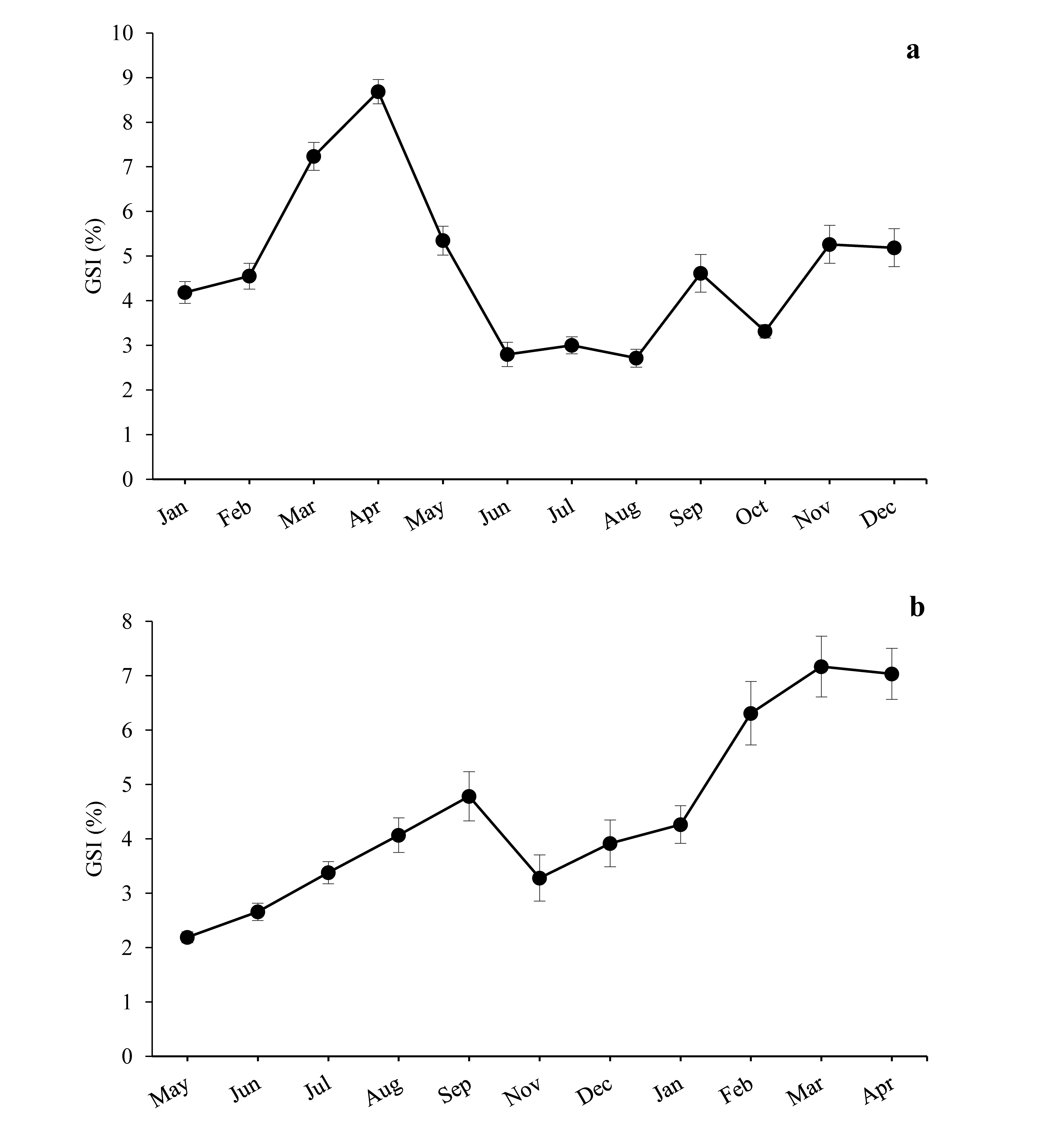

Supplement: Supplemental Information 7 — GSI trend of sea urchin population at (A) a location (Seu) in the Sinis peninsula, contiguous to Su Pallosu Bay (Baroli et al., 2006), and (B) Tavolara—Punta Coda Cavallo (Guala, Simeone & Baroli, 2009). [file peerj-05-3067-s007.jpg]
